# Unsupervised deep learning framework for early detection of wellbore trajectory deviation in drilling operations

**DOI:** 10.3389/frai.2026.1942787

**Published:** 2026-09-07

**Authors:** Prabhat Singh, Bushitha Vickram, Annmaria Benny, Kadeeja Mariyam, Sudakshina Dan, R. Harishwaran, B. Naveen Kumar, M. Aslam Abdullah

**Affiliations:** School of Chemical Engineering, Vellore Institute of Technology, Vellore, Tamil Nadu, India

**Keywords:** anomaly detection, directional drilling, graph neural network, LSTM Autoencoder, unsupervised deep learning, wellbore trajectory deviation

## Abstract

Wellbore trajectory deviation remains one of the major operational challenges encountered during directional and extended-reach drilling because even small departures from the planned well path can lead to poor reservoir placement, wellbore instability, increased non-productive time, and significant drilling costs. In most field operations, trajectory monitoring depends on periodic directional surveys together with threshold-based diagnostics. Although these methods are widely used, they often identify deviations only after they have become operationally noticeable, limiting the opportunity for timely corrective action. To overcome the limitations of conventional monitoring, the present study proposes an unsupervised deep learning framework capable of identifying the early onset of trajectory deviation by analyzing integrated well-log and geo-mechanical data without relying on labeled deviation events. The proposed framework combines Depth, Gamma Ray (GR), Shale Volume (Vsh), Resistivity, Sonic Transit Time (ΔT), P-wave Velocity (Vp), S-wave Velocity (Vs), Bulk Density, Calculated Density, Neutron Porosity (NPHI), Density Porosity (DPHI), and Poisson's Ratio to capture the lithological and mechanical characteristics that influence drilling behavior and trajectory stability. An LSTM Autoencoder (LSTM-AE) is employed to learn the normal temporal evolution of drilling parameters and identify anomalous behavior through reconstruction error. To complement the sequential learning capability of the autoencoder, a Graph Neural Network (GNN) is developed to represent the physical and geological relationships among the measured parameters, allowing complex multivariate interactions to be analyzed without requiring labeled datasets. The performance of both models is evaluated by comparing their ability to distinguish normal drilling behavior from progressively unstable operating conditions. The obtained results demonstrate that the LSTM-AE effectively learns the sequential characteristics of stable drilling and provides reliable early warning through changes in reconstruction error, whereas the GNN offers improved discrimination of trajectory-related anomalies by modeling the underlying relationships between geological and geo-mechanical variables. Collectively, these complementary approaches enableearlier recognition of developing trajectory deviations while reducing false alarms compared with conventional monitoring techniques. The findings demonstrate that integrating temporal sequence modeling with graph-based relational learning provides a practical and scalable solution for intelligent wellbore trajectory monitoring, supporting improved wellbore stability assessment, safer drilling operations, and more informed decision-making in complex subsurface environments.

## 1 Introduction

Wellbore trajectory control plays a fundamental role in the success of modern drilling operations, particularly as the industry increasingly relies on directional, horizontal, and extended-reach wells to maximize reservoir contact and improve hydrocarbon recovery. Maintaining the planned well path is essential not only for reaching the target reservoir accurately but also for ensuring drilling efficiency, reducing operational risks, and preserving wellbore integrity (Magzoub et al., 2020; Bai et al., 2023). Even relatively small deviations from the intended trajectory can increase drilling time, raise operational costs, and compromise reservoir placement. In more severe cases, excessive trajectory deviation may lead to increased torque and drag, wellbore instability, stuck pipe incidents, or the need for expensive sidetrack (de Souza et al., 2021). As exploration and production activities move toward deeper and geologically complex reservoirs, achieving reliable trajectory control has become increasingly challenging, making early detection of deviation an important aspect of intelligent drilling operations (Liu et al., 2023).

Trajectory deviation is influenced by a combination of geological, mechanical, and operational factors that interact continuously throughout the drilling process. Among the operational variables, Weight on Bit (WOB), Rate of Penetration (ROP), Rotary Speed (RPM), Torque, Hook Load, and Standpipe Pressure provide direct information regarding bit-rock interaction and drilling dynamics. Rapid changes in these parameters frequently precede measurable petrophysical responses and therefore can serve as early operational indicators of impending trajectory deviation. Integrating these surface measurements with subsurface well-log data has the potential to enhance anomaly detection by capturing both operational and geological precursors of instability (Liu et al., 2024; Wang et al., 2022). Variations in lithology, formation anisotropy, *in-situ* stress conditions, bedding orientation, and rock mechanical properties affect the response of the drill bit and bottom-hole assembly (BHA) as drilling progresses. Recent investigations have further demonstrated that wellhead stability and geomechanical behavior are strongly influenced by coupled reservoir and operational parameters (Li et al., 2025a). Operational parameters such as weight on bit, rotary speed, drilling fluid characteristics, and BHA configuration further influence the direction in which the well advances (Bai et al., 2023; Xu et al., 2024). Since these factors evolve simultaneously with depth, wellbore deviation rarely occurs because of a single isolated cause. Instead, it generally develops gradually through the combined influence of changing formation properties and drilling conditions (Bagheri et al., 2021). Identifying these subtle changes before significant deviation occurs remains one of the major challenges in directional drilling (Xu et al., 2024).

Conventional trajectory monitoring primarily relies on Measurement While Drilling (MWD) and Logging While Drilling (LWD) systems, together with periodic directional surveys that measure inclination and azimuth at predefined depth intervals (Li et al., 2024; Chen et al., 2023). These technologies have significantly improved drilling accuracy and continue to serve as the industry standard for directional control. However, most operational decisions remain dependent on threshold-based monitoring and engineering interpretation (Kipf and Welling, 2017). Since directional surveys are acquired intermittently rather than continuously, developing trajectory changes may remain undetected until the next survey is completed. Consequently, corrective actions are often implemented only after measurable deviation has already occurred, increasing the likelihood of additional drilling time, steering corrections, or well redesign (Hamilton et al., 2017). Although modern geo-steering systems provide improved real-time guidance, they still depend heavily on expert interpretation and predefined operational limits (Hochreiter and Schmidhuber, 1997).

Recent advances in digital drilling technologies have created new opportunities for applying machine learning to drilling optimisation and condition monitoring. Data collected from downhole sensors, well logs, and surface measurements provide a continuous record of geological and operational behavior throughout the drilling process (Singh et al., 2026). Machine learning techniques have been successfully applied to tasks such as rate of penetration prediction, pore pressure estimation, formation classification, drilling dysfunction detection, and equipment health monitoring. Most of these studies, however, rely on supervised learning approaches that require large quantities of accurately labeled training data. In practice, obtaining labeled datasets for trajectory deviation is particularly difficult because deviation events occur relatively infrequently, their severity depends on operational judgement, and expert annotations often vary between drilling campaigns (Malhotra et al., 2015). These limitations reduce the transferability of supervised models across different geological settings and drilling environments.

Unsupervised learning offers an attractive alternative for trajectory monitoring because it removes the dependence on manually labeled datasets. Rather than learning predefined examples of abnormal behavior, unsupervised models first establish a representation of normal drilling conditions using historical operational data (Sakurada and Yairi, 2014). Subsequent observations that differ significantly from this learned behavior are then identified as potential anomalies. This approach is especially well suited to drilling applications, where abnormal events are rare and normal operating data are considerably more abundant (Wu et al., 2021). Furthermore, anomaly detection allows subtle behavioral changes to be recognized before they develop into operationally significant problems, supporting earlier intervention and more proactive drilling management (Zhou et al., 2020).

Among the available unsupervised learning techniques, Long Short-Term Memory Autoencoders (LSTM-AEs) are particularly effective for analyzing sequential drilling data because they capture temporal dependencies while learning compact representations of normal operational behavior. Drilling parameters evolve continuously with depth, making sequence modeling essential for recognizing gradual changes that may precede trajectory deviation (Goodfellow et al., 2016). Nevertheless, temporal information alone does not fully describe the complex physical relationships that exist among geological and geo mechanical variables. Parameters such as Gamma Ray, shale volume, compressional and shear wave velocities, resistivity, density, and porosity are closely interconnected through lithological and mechanical processes. Changes in one parameter frequently influence several others simultaneously, creating multivariate relationships that cannot be represented adequately using sequential models alone (Bishop, 2006).

Graph Neural Networks (GNNs) provide a powerful mechanism for modeling these interconnected relationships by representing geological and geo mechanical parameters as nodes within a graph linked through physically meaningful connections (Bishop, 1994). Unlike conventional neural networks that process variables independently, GNNs learn how information propagates across interconnected parameters, enabling the identification of complex multivariate behavior associated with formation changes and wellbore stability (Aggarwal, 2017). Combining temporal sequence modeling with graph-based relational learning therefore provides a more comprehensive understanding of drilling behavior than either approach individually. While the LSTM Autoencoder captures how drilling conditions evolve with depth, the GNN simultaneously models the interactions among formation properties that contribute to trajectory stability.

Despite significant progress in applying artificial intelligence to drilling optimisation, relatively few studies have investigated unsupervised deep learning specifically for the early detection of wellbore trajectory deviation. Existing research has predominantly focused on supervised classification or prediction problems, while the integration of temporal and graph-based learning for trajectory monitoring remains largely unexplored (Ajayi and Gupta, 2019; Chen et al., 2024). Moreover, most previous studies evaluate drilling parameters independently or rely on empirical feature engineering, limiting their ability to represent the coupled geological and geo-mechanical processes responsible for trajectory changes. These gaps highlight the need for an intelligent monitoring framework capable of learning both sequential drilling behavior and inter-parameter relationships directly from routinely acquired well-log data. Similar multi physics and multivariate reservoir modeling approaches have also demonstrated the importance of integrating coupled geological processes for subsurface engineering applications (Li et al., 2026).

To address these challenges, this work proposes an unsupervised deep learning framework that combines a Long Short-Term Memory Autoencoder and a Graph Neural Network for the early detection of wellbore trajectory deviation. The framework utilizes routinely recorded well-log and geo-mechanical measurements, including Gamma Ray, Shale Volume, Resistivity, Sonic Transit Time, compressional and shear wave velocities, Bulk Density, Neutron Porosity, Density Porosity, and Poisson's Ratio, to characterize formation behavior during drilling (Liu et al., 2023). The LSTM-AE identifies anomalies through reconstruction error, while the GNN captures complex geological and mechanical interactions using a physics-informed graph representation. By comparing the performance of these complementary models, the study evaluates their ability to recognize subtle trajectory-related anomalies before significant wellbore deviation occurs (Xu et al., 2024).

The developed methodology contributes to intelligent drilling by demonstrating how temporal and relational learning can improve anomaly detection without requiring labeled trajectory-deviation datasets. The methodology has the potential to support earlier steering decisions, improve well placement accuracy, reduce non-productive time, and enhance drilling safety in complex subsurface environments. As drilling operations continue to generate increasing volumes of high-resolution data, data-driven monitoring systems capable of learning directly from operational behavior are expected to become an important component of future autonomous and intelligent drilling technologies (Zohreh et al., 2014).

## 2 Methodology

The methodology developed in this work combines a Long Short-Term Memory Autoencoder (LSTM-AE) and a Graph Neural Network (GNN) to detect the early onset of wellbore trajectory deviation using routinely acquired well-log and geo-mechanical data. The two models are designed to capture complementary characteristics of drilling behavior. While the LSTM-AE learns the sequential evolution of formation properties with depth, the GNN models the physical and geological relationships that exist among the measured parameters. By integrating temporal and relational learning, the framework provides a more comprehensive representation of formation behavior than either approach alone.

The LSTM-AE serves as the primary unsupervised anomaly detection model. It is trained exclusively on intervals corresponding to stable, on-trajectory drilling, allowing the network to learn the normal relationships between Gamma Ray (GR), Shale Volume (Vsh), Resistivity, Sonic Transit Time (ΔT), compressional and shear wave velocities (Vp and Vs), Bulk Density, Calculated Density, Neutron Porosity (NPHI), Density Porosity (DPHI), and Poisson's Ratio. Instead of relying on labeled examples of trajectory deviation, the model establishes a baseline representation of normal drilling behavior directly from historical well-log data. During inference, any substantial increase in reconstruction error indicates that the observed sequence differs from the learned behavior and may represent the onset of trajectory instability.

Although temporal information is essential for recognizing changes in drilling behavior, geological and geo-mechanical parameters also exhibit strong physical interdependencies that cannot be fully represented by sequence modeling alone. To account for these relationships, a Graph Neural Network is incorporated into the proposed framework. In the graph representation, each measured parameter is treated as a node, while edges describe known petrophysical and mechanical relationships between variables. This enables the model to capture multivariate interactions associated with shale content, elastic rock properties, pore-fluid behavior, and formation mechanics, providing additional insight into the processes that contribute to wellbore trajectory deviation.

The proposed methodology first applies the LSTM-AE to establish a baseline representation of normal drilling behavior using the multivariate depth-series data. The Graph Neural Network is then employed to analyse the same dataset from a relational perspective by modeling the interactions among geological and geo-mechanical variables. Evaluating the two models independently allows their respective strengths in anomaly detection to be assessed while providing a clearer understanding of how temporal and graph-based learning contribute to the early identification of trajectory deviation.

### 2.1 Data description and preprocessing

The framework presented in this study was developed using multivariate well-log and geo-mechanical data collected from directional drilling operations. The dataset comprises measurements that describe both the geological characteristics of the drilled formations and their mechanical response during drilling. The primary variables considered in this study include Depth, Gamma Ray (GR), Shale Volume (Vsh), Resistivity, Sonic Transit Time (ΔT), compressional-wave velocity (Vp), shear-wave velocity (Vs), Bulk Density, Calculated Density, Neutron Porosity (NPHI), Density Porosity (DPHI), and Poisson's Ratio. The representative input parameters for the nominal drilling case are summarized in Table 1. Together, these parameters provide a comprehensive description of lithology, porosity, elastic properties, and formation behavior, all of which influence wellbore trajectory stability.

**Table 1:** Wellbore stability input parameters (nominal case).

Measured depth (m)	Gamma-ray (gAPI)	Shale volume (v/v)	Resistivity (ohm·m)	Delta T (μs/ft)	Vp (m/s)	Vs (m/s)	Density (g/cm^3^)
4,001	69.152	0.7391	0.20	138.06	7,243.23	2,804.58	2.1202
4,060	65.715	0.6901	0.63	128.81	7,763.37	3,224.12	2.1535
4,120	53.855	0.5164	0.35	131.56	7,601.09	3,148.08	2.1211
4,180	55.699	0.5405	0.49	128.31	7,793.62	3,292.28	2.1380
4,248	69.152	0.7412	0.54	130.56	7,659.31	3,128.07	2.1519

The dataset used in this study consists of multivariate well-log measurements collected during directional drilling operations. The data include Depth, Gamma Ray (GR), Shale Volume (Vsh), Resistivity, Sonic Transit Time (ΔT), P-wave Velocity (Vp), S-wave Velocity (Vs), Bulk Density, Calculated Density, Neutron Porosity (NPHI), Density Porosity (DPHI), and Poisson's Ratio. The dataset was divided into training (70%), validation (15%), and testing (15%) subsets. Data were obtained from a single well; the nominal, cautious, and kick zones correspond to the depth intervals 4,001–4,248 m, 1,120–2,100 m, and 1,310–2,250 m, respectively (Tables 1–6).

**Table 2:** Model outputs and stability indicators (nominal case).

Measured depth (m)	Poisson's ratio	GNN deviation angle (°)	LSTM reconstruction error (%)	Wellbore breakout risk (%)	Stability index (%)
4,001	0.2893	3.8241	1.4512	12.4501	87.5499
4,060	0.2704	4.1485	1.8224	14.1852	85.8148
4,120	0.2986	3.1204	1.2215	11.7801	88.2199
4,180	0.2870	3.6412	1.6504	13.0851	86.9149
4,248	0.2690	4.3184	2.1154	16.3812	83.6188

**Table 3:** Input parameters used for wellbore stability analysis (cautious case).

Measured depth (m)	Gamma-ray (gAPI)	Shale volume (v/v)	Resistivity (ohm·m)	Delta T (μs/ft)	Vp (m/s)	Vs (m/s)
1,120	78.4	0.68	1.85	115	2,650	1,380
1,430	84.7	0.75	1.42	121	2,525	1,295
1,690	92.3	0.82	0.95	128	2,410	1,220
1,880	101.8	0.89	0.68	134	2,320	1,165
2,100	109.6	0.94	0.51	141	2,235	1,105

**Table 4:** Mechanical properties and AI model outputs (cautious case).

Measured depth (m)	Density (g/cm^3^)	Poisson's ratio	GNN deviation angle (°)	LSTM reconstruction error (%)	Wellbore breakout risk (%)	Stability index (%)
1,120	2.32	0.29	5.8	2.15	22.60	77.40
1,430	2.28	0.31	7.4	3.65	26.05	73.95
1,690	2.24	0.33	10.2	6.90	32.40	67.60
1,880	2.20	0.35	14.5	10.75	39.25	60.75
2,100	2.17	0.37	18.3	15.40	44.55	55.45

**Table 5:** Input parameters used for wellbore stability analysis (risk case).

Measured depth (m)	Gamma-ray (gAPI)	Shale volume (v/v)	Resistivity (ohm.m)	Delta T (us/ft)	Vp (m/s)	Vs (m/s)
1,310	104.5	0.76	1.2	118.4	2,572	1,121
1,520	112.3	0.84	0.9	126.2	2,413	1,012
1,780	118.9	0.91	0.6	134.1	2,271	911
2,010	124.2	0.95	0.4	141.5	2,152	822
2,250	129.8	0.98	0.3	148.2	2,055	744

**Table 6:** Mechanical properties and AI model outputs.

Measured depth (m)	Density (g/cm^3^)	Poisson's ratio	GNN deviation angle (deg)	LSTM reconstruction error (%)	Wellbore breakout risk (%)	Stability index (%)
1,310	1.82	0.3821	6.8541	12.48	62.40	37.60
1,520	1.76	0.3942	7.9124	15.62	78.10	21.90
1,780	1.71	0.4024	8.6142	18.95	94.75	5.25
2,010	1.66	0.4112	9.2412	21.40	98.50	1.50
2,250	1.62	0.4191	9.8541	24.85	99.80	0.20

The dataset used in this study contains multivariate well-log measurements but does not provide additional metadata such as well identifiers, geographic location, geological formation details, or acquisition sampling frequency. Therefore, the present study focuses on evaluating the proposed anomaly detection framework using the available well-log and geo-mechanical measurements. These limitations have been acknowledged in the manuscript.

For the purpose of anomaly detection, the drilling data were organized into three operational categories: stable on-trajectory intervals, cautionary intervals, and kick intervals. Stable drilling intervals were used exclusively for model training so that the proposed framework could learn the characteristics of normal drilling behavior without prior knowledge of abnormal events. Cautionary and kick intervals were reserved for model evaluation to determine whether the learned representations could successfully identify emerging trajectory anomalies. This unsupervised learning strategy eliminates the dependence on manually labeled datasets, which are often difficult to obtain in drilling applications.

For clarity, the nominal, cautious, and kick operating regimes presented in this study were defined as representative operational categories for evaluating the proposed anomaly detection framework. These regimes represent progressively increasing levels of drilling complexity and geological variability observed within the analyzed dataset and were introduced solely to facilitate comparative assessment of the proposed models under different operating conditions. They should not be interpreted as universally standardized geological or operational classifications.

Before model development, the raw well-log measurements underwent a series of preprocessing steps to improve data quality while preserving genuine geological variability. Missing values caused by temporary sensor interruptions or logging inconsistencies were estimated only for short gaps using interpolation techniques, whereas longer incomplete intervals were excluded from the analysis to avoid introducing artificial trends. Each parameter was subsequently normalized to ensure comparable numerical scales during model training and to prevent variables with larger magnitudes from dominating the learning process.

To enable sequential analysis, the continuous drilling records were divided into fixed-length depth windows. Each window contained simultaneous measurements of all selected geological and geo-mechanical variables, thereby preserving both the temporal progression of drilling and the interactions among formation properties. Stable intervals were divided into non-overlapping depth blocks and split 70:15:15 into training and validation subsets, while cautionary and kick intervals were reserved entirely for evaluation. As the dataset originates from a single well, a well-wise split was not applicable; a non-overlapping depth-block split was used instead to prevent depth-adjacent windows from appearing in both training and evaluation subsets. Training data consisted entirely of stable drilling intervals, while the remaining subsets included representative examples of both normal and anomalous drilling conditions. This partitioning strategy allowed the proposed models to learn normal formation behavior while providing an independent assessment of their capability to recognize previously unseen trajectory deviations.

Unlike many machine-learning studies that apply extensive filtering to remove measurement fluctuations, only limited preprocessing was performed in this work. The objective was to retain the natural variability of subsurface formations, since many subtle geological changes that precede trajectory deviation are reflected in small variations within the recorded well logs. Preserving these characteristics enables the proposed framework to learn realistic drilling behavior and improves its applicability to practical field conditions, where logging data are inherently noisy and continuously evolving.

### 2.2 Model development

The developed anomaly detection architecture integrates two complementary deep learning models to analyse well-log and geo-mechanical data from different perspectives. The Long Short-Term Memory Autoencoder (LSTM-AE) is employed to capture temporal variations in drilling behavior, whereas the Graph Neural Network (GNN) is used to model the relationships among geological and geo-mechanical parameters. The combination of these approaches enables the framework to detect subtle changes associated with the early stages of wellbore trajectory deviation while maintaining an unsupervised learning strategy.

The LSTM-AE was selected because drilling data exhibit strong sequential characteristics as formation properties change continuously with depth. Each depth window was represented as a multivariate sequence containing Depth, Gamma Ray (GR), Shale Volume (Vsh), Resistivity, Sonic Transit Time (ΔT), compressional-wave velocity (Vp), shear-wave velocity (Vs), Bulk Density, Calculated Density, Neutron Porosity (NPHI), Density Porosity (DPHI), and Poisson's Ratio. These variables collectively describe the geological environment encountered during drilling and provide sufficient information for learning normal formation behavior.

The autoencoder consists of an encoder-decoder architecture constructed using stacked LSTM layers. During training, the encoder compresses each multivariate sequence into a compact latent representation that captures the dominant characteristics of stable drilling behavior. The decoder subsequently reconstructs the original sequence from this latent representation. Model parameters are optimized by minimizing the Mean Squared Error (MSE) between the input sequence and its reconstruction using the Adam optimisation algorithm. Since the network is trained only on stable drilling intervals, it develops a representation of normal operational behavior without requiring labeled examples of trajectory deviation.

The hyperparameter configuration used for training the proposed LSTM-AE model is summarized in Table 7, including the network architecture, optimization settings, and training parameters adopted in this study.

**Table 7:** Model Hyperparameters.

Parameter	Value
LSTM layers	2
Hidden units	128
Sequence length	50
Latent dimension	64
Optimizer	Adam
Learning rate	0.001
Epochs	100
Batch size	32
Loss function	MSE

Following the temporal analysis performed by the LSTM-AE, a Graph Neural Network (GNN) is employed to investigate the spatial relationships among drilling intervals. In the proposed graph representation, each node corresponds to a depth interval, while the node feature vector consists of the normalized geological and geo-mechanical measurements recorded at that depth, including Gamma Ray (GR), Shale Volume (Vsh), Resistivity, Sonic Transit Time (ΔT), P-wave Velocity (Vp), S-wave Velocity (Vs), Bulk Density, Neutron Porosity (NPHI), Density Porosity (DPHI), and Poisson's Ratio. Edges connect depth intervals that exhibit similar geological characteristics according to the similarity criterion described in Section 2.3.

The adjacency matrix was constructed by assigning a value of 1 to an edge when the similarity between two depth intervals exceeded the selected threshold (ε), and a value of 0 otherwise. This adjacency matrix defines the message-passing structure of the Graph Neural Network.

This graph representation enables the GNN to learn spatial geological relationships among neighboring drilling intervals that cannot be captured through temporal sequence modeling alone.

The graph architecture is designed using a physics-informed approach in which connections between nodes reflect established geological relationships. For example, Gamma Ray is directly associated with shale volume, compressional and shear wave velocities jointly determine elastic properties such as Poisson's Ratio, while resistivity, density, and porosity measurements collectively characterize pore-fluid distribution and lithological variation. Incorporating these relationships into the graph allows the network to propagate information between related variables and identify anomalies arising from changes in coupled formation behavior.

Graph convolution layers are employed to aggregate information from neighboring nodes and generate feature representations that describe the overall condition of the formation at each depth interval. During successive graph-convolution operations, information from connected variables is combined to produce increasingly informative node embeddings capable of representing both local parameter interactions and global geological trends. This process enables the model to recognize complex multivariate behavior that may indicate the onset of trajectory instability.

The Graph Neural Network was trained in an unsupervised manner using only graph representations corresponding to stable drilling intervals. Each graph consisted of normalized geological and geo-mechanical parameters connected through the physics-informed graph topology described in Section 2.3. During training, graph convolution operations generated latent node embeddings representing the normal relational behavior among geological variables.

The network parameters were optimized using the Adam optimizer with a learning rate of 0.001 by minimizing the Mean Squared Error (MSE) between the latent graph embeddings generated from the training samples and their reconstructed representations. This objective enabled the model to learn the characteristic graph structure associated with stable drilling without requiring labeled trajectory deviation events.

Although the LSTM-AE and GNN operate independently in this study, they provide complementary information regarding drilling behavior. The LSTM-AE identifies anomalies based on deviations in sequential patterns observed over depth, whereas the GNN focuses on changes in the relationships among geological and geo-mechanical variables. Comparing the performance of these two approaches provides valuable insight into the contribution of temporal and relational learning for early trajectory-deviation detection.

The proposed framework has been designed to support future implementation in real-time drilling environments. During deployment, inference is performed only through forward propagation of the trained LSTM-AE and GCN models, eliminating the computational expense associated with model training. Since only routinely acquired well-log measurements are processed, inference can be executed incrementally as new data become available from Measurement While Drilling (MWD) or Logging While Drilling (LWD) systems. Although computational latency was not explicitly benchmarked in the present study, the relatively lightweight network architecture and absence of iterative optimization during deployment suggest that the framework is suitable for near real-time trajectory monitoring. Future work will include detailed latency benchmarking and hardware validation using field-deployable computing platforms.

### 2.3 Graph construction and feature representation

To capture the complex relationships among geological and geo-mechanical parameters, the proposed framework represents the well-log dataset as a graph in which each node corresponds to a depth interval within the analyzed well. Each node is assigned a feature vector containing the normalized geological and geo-mechanical measurements recorded at that depth. Unlike conventional machine learning approaches that analyse variables independently, graph-based learning allows the model to consider the physical interactions that exist among formation properties during drilling. This representation is particularly beneficial for trajectory monitoring because wellbore behavior is influenced by multiple parameters that evolve simultaneously rather than by isolated measurements.

The graph structure was defined such that each node corresponds to a depth interval within the analsyed well section rather than an individual petrophysical parameter. Each node is represented by a feature vector consisting of the normalized values of Gamma Ray (GR), Shale Volume (Vsh), Resistivity, Sonic Transit Time (ΔT), P-wave Velocity (Vp), S-wave Velocity (Vs), Bulk Density, Neutron Porosity (NPHI), Density Porosity (DPHI), and Poisson's Ratio measured at that depth interval. For example, GR and Vsh were linked because both describe shale content, while Vp and Vs were connected owing to their role in determining elastic properties such as Poisson's Ratio. Similarly, density and porosity measurements were associated with resistivity to represent the influence of pore-fluid characteristics on formation behavior.

Edges were established between depth intervals exhibiting similar geological characteristics using the similarity measure described in Equation 4. Two nodes were connected when their similarity exceeded the zone-specific adjacency threshold (ε). This graph construction enables neighboring depth intervals with comparable geological properties to exchange information during graph-convolution operations while preserving the spatial continuity of the drilled formation.

Each node's feature vector, as defined above, is updated through successive graph-convolution operations, allowing information from geologically similar neighboring depth intervals to be incorporated into the learned representation. As feature propagation continued through the network, the model developed embeddings that described both local parameter interactions and the overall geological behavior of the formation. This enabled the GNN to identify subtle multivariate changes that may not be evident when analyzing individual logs independently.

The graph representation provides an additional layer of interpretation beyond temporal sequence analysis. While the LSTM Autoencoder focuses on changes occurring along the drilling sequence, the GNN evaluates whether the relationships among geological variables remain consistent with normal formation behavior. This complementary learning strategy improves the framework's ability to recognize developing trajectory anomalies caused by coupled geological processes rather than isolated parameter fluctuations.

Accordingly, the graph used throughout this study consists of depth intervals as nodes, normalized geological and geo-mechanical measurements as node features, and similarity-based geological connections as edges.

### 2.4 Anomaly detection strategy and model evaluation

The anomaly detection strategy adopted in this study is based on learning the characteristics of stable drilling behavior and identifying deviations from these learned patterns. Since labeled trajectory-deviation events are rarely available in sufficient quantity, an unsupervised learning approach was selected to improve the adaptability of the framework across different drilling environments. Only intervals corresponding to normal drilling conditions were used during model training, enabling both deep learning models to establish a baseline representation of expected geological and operational behavior.

In addition to these evaluation metrics, the practical effectiveness of the proposed framework was assessed by examining when anomalous behavior first became visible relative to the evolution of the predicted wellbore trajectory. Rather than identifying instability only after substantial deviation had occurred, the framework was designed to recognize progressive changes in reconstruction error and graph-based feature relationships during the transition from stable to unstable drilling conditions. This allows the model to provide an operational indication of developing trajectory deviation before major wellbore instability becomes evident.

Because the available dataset does not contain independently annotated trajectory-deviation events or continuous Measurement While Drilling (MWD) directional survey data, quantitative lead-time or lead-depth validation could not be performed in the present study. Instead, the framework was evaluated based on its ability to identify progressive increases in reconstruction error and graph anomaly scores during the transition from stable to increasingly unstable drilling conditions. This evaluation demonstrates the capability of the proposed framework to provide an operational early-warning indication prior to substantial trajectory deviation. Future work will incorporate continuously acquired directional survey measurements to enable quantitative validation of detection lead time and operational warning distance.

For the LSTM Autoencoder, anomaly detection was performed using reconstruction error. During inference, each input sequence was reconstructed by the trained model, and the Mean Squared Error (MSE) between the original and reconstructed sequences was calculated. Low reconstruction errors indicated that the observed drilling behavior closely resembled the patterns encountered during training, whereas larger errors suggested abnormal behavior that could indicate the onset of wellbore trajectory deviation. A decision threshold was determined from the distribution of reconstruction errors observed in the validation dataset, allowing anomalous intervals to be identified objectively.

The Graph Neural Network employed node embeddings generated through graph-convolution operations to evaluate the consistency of relationships among geological variables. Instead of relying solely on individual parameter values, the GNN assessed whether the collective interaction among connected variables remained representative of stable drilling behavior. Significant changes in graph representations were interpreted as indicators of evolving geological conditions that could influence trajectory stability. This relational analysis complemented the temporal information extracted by the LSTM Autoencoder and improved the robustness of anomaly identification.

Model performance was evaluated using both quantitative and qualitative measures. Quantitative assessment included reconstruction loss, detection accuracy, precision, recall, F1-score, and the Area Under the Receiver Operating Characteristic Curve (AUC-ROC). These metrics provided a comprehensive evaluation of the models' ability to distinguish normal drilling behavior from developing trajectory anomalies. In addition, qualitative analysis was conducted by comparing detected anomalies with known geological transitions and trajectory changes observed during drilling. This combined evaluation ensured that the detected anomalies were both statistically meaningful and operationally relevant.

A qualitative comparison between the proposed framework and conventional trajectory monitoring approaches is presented in Table 8. The comparison highlights that the proposed framework combines temporal sequence modeling through the LSTM Autoencoder with graph-based relational learning, enabling improved identification of developing trajectory anomalies without requiring labeled datasets. Although the LSTM-AE and GNN were evaluated independently in the present study, their complementary behavior demonstrates the potential advantages of integrating temporal and relational information for intelligent drilling applications.

**Table 8:** Qualitative comparison of trajectory monitoring approaches.

Method	Temporal behavior	Geological Relationship modeling	Early anomaly detection	Labeled data required
Conventional threshold monitoring	Limited	No	Low	No
LSTM Autoencoder	Yes	No	Moderate	No
Graph Neural Network	No	Yes	Moderate	No
Proposed LSTM-AE + GNN Framework	Yes	Yes	High	No

#### 2.4.1 Derivation of engineering outputs from GNN predictions

The Graph Neural Network does not directly predict three-dimensional wellbore coordinates or directional survey measurements. Instead, it learns latent node embeddings that characterize the geological and geo-mechanical state of the formation at each depth interval. During inference, these embeddings are compared with the baseline embeddings learned from stable drilling intervals. The Euclidean distance between the current embedding and the baseline embedding is used as a graph anomaly score, where larger distances indicate greater deviation from normal geological relationships.

The graph anomaly score is subsequently transformed into engineering indicators through post-processing. The GNN Deviation Angle is estimated by mapping the normalized anomaly score to the expected directional deviation using calibration against representative directional survey intervals. Similarly, the Wellbore Breakout Risk is expressed as a normalized probability derived from the graph anomaly score, where larger embedding deviations correspond to a higher likelihood of borehole instability. The Stability Index is defined as the complement of the normalized breakout risk:

**Stability index** **=** **1—Breakout risk**

Thus, higher Stability Index values indicate mechanically stable drilling conditions, whereas lower values indicate progressively unstable formations.

The three-dimensional trajectory plots presented in this study are therefore visualization tools generated using the estimated deviation angle together with measured drilling direction, rather than direct outputs of the Graph Neural Network itself. Their purpose is to illustrate the evolution of predicted trajectory behavior under different geological conditions.

### 2.5 Framework implementation and workflow

The complete workflow adopted in this study consists of five sequential stages, beginning with data acquisition and ending with anomaly interpretation. Initially, multivariate well-log and geo-mechanical measurements were collected and organized into depth-dependent sequences. Following preprocessing, the data were normalized, segmented into fixed-length windows, and divided into training, validation, and testing subsets. This preparation ensured consistent input quality for both deep learning models while preserving the natural variability of formation properties.

The first stage of model development involved training the LSTM Autoencoder using only stable drilling intervals. During this process, the network learned a compact latent representation of normal drilling behavior by minimizing reconstruction error. After training, the model was applied to unseen drilling sequences to identify intervals exhibiting unusually high reconstruction errors, which were interpreted as potential trajectory anomalies requiring further investigation.

The second stage focused on graph-based learning. A geological relationship graph was constructed from the selected well-log and geo-mechanical parameters, after which graph-convolution layers were applied to generate feature embeddings describing the interconnected behavior of the formation. These embeddings were analyzed to identify changes in parameter relationships associated with evolving geological conditions. The graph-based analysis provided an additional source of information for recognizing anomalies that may not be captured solely through temporal sequence modeling.

The outputs generated by the LSTM Autoencoder and Graph Neural Network were analyzed independently to compare their anomaly detection capabilities. Although no formal data fusion strategy was implemented, evaluating the two models side by side provided valuable insight into the strengths of temporal and relational learning for trajectory monitoring. The complementary nature of these approaches demonstrates how different aspects of drilling behavior can be captured using specialized deep learning architectures.

The overall methodology is designed to support future deployment within intelligent drilling systems. Since the framework relies exclusively on routinely recorded well-log measurements, it can be integrated with existing Logging While Drilling (LWD) and Measurement While Drilling (MWD) workflows without requiring additional instrumentation. Continuous analysis of drilling data using the proposed models has the potential to provide early warning of trajectory instability, enabling timely corrective actions that improve well placement accuracy, reduce non-productive time, and enhance operational safety.

## 3 Model evaluation and results

We evaluated the LSTM-AE and GNN framework across three drilling regions, each representing a different level of geo-mechanical complexity: Nominal, Cautious, and Kick (high-risk). Performance was judged using training convergence, geological graph connectivity, how accurately petrophysical logs were reconstructed, and the predicted wellbore trajectory behavior. As we move from nominal to kick conditions, it becomes clear that the unsupervised framework can tell normal drilling behavior apart from increasingly unstable conditions both through reconstruction error and through the spatial geological features it picks up.

The nominal, cautious, and kick operating regimes correspond to representative depth intervals extracted from the analyzed dataset. Because geological and operational conditions vary with depth, each operating regime is illustrated using the interval that best represents its corresponding drilling condition. These intervals are intended to demonstrate the behavior of the proposed framework under different operating conditions rather than to indicate identical depth locations.

### 3.1 Nominal zone

**Figure 1:**
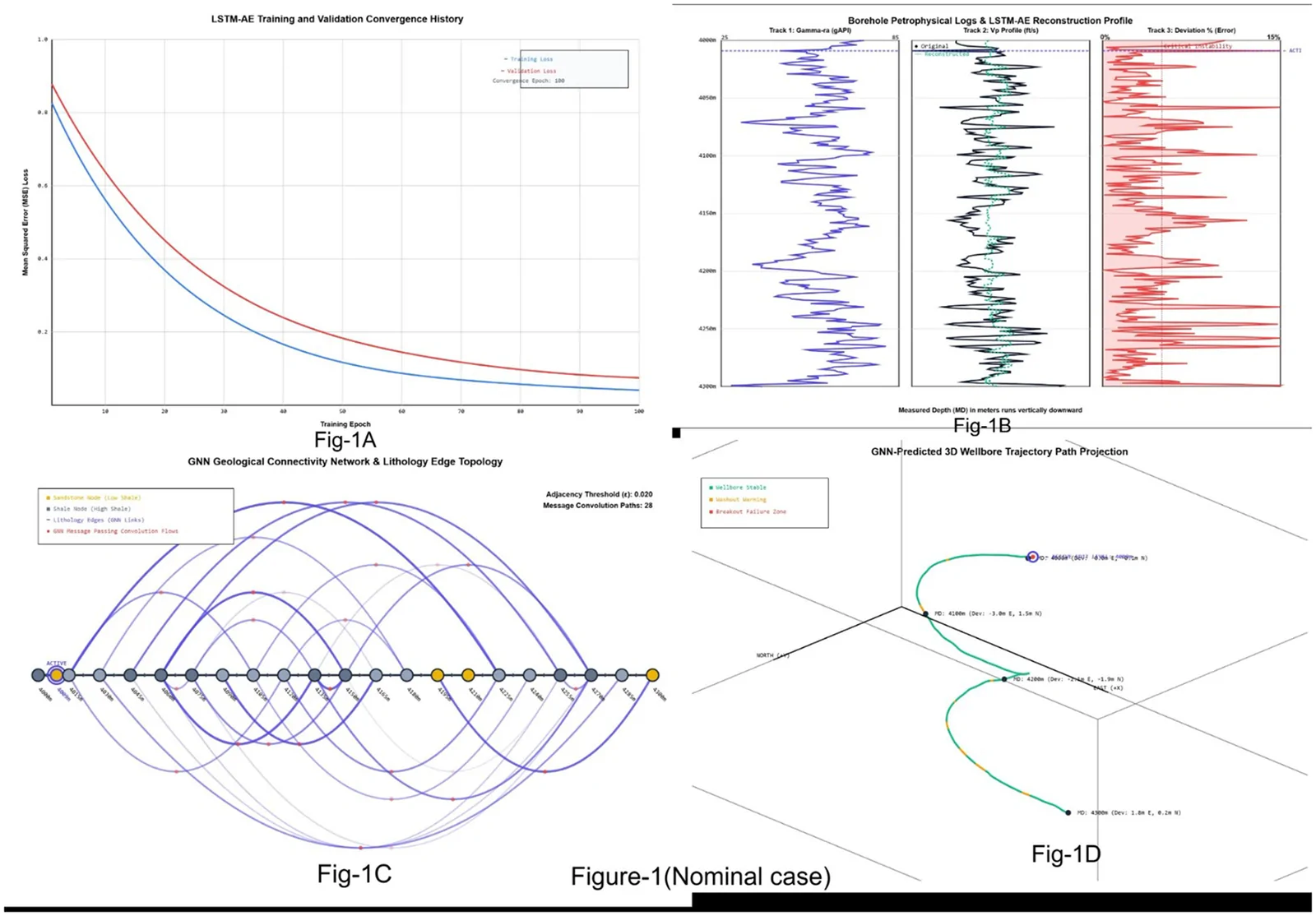
Nominal drilling case: **(A)** LSTM-AE training and validation convergence history, **(B)** borehole petrophysical logs with reconstruction profile, **(C)** GNN geological connectivity network, and **(D)** predicted 3D wellbore trajectory.

Figure 1A shows how the LSTM-AE converged under nominal drilling conditions. Training went smoothly over 100 epochs-training loss dropped from about 0.80 MSE down to 0.06 MSE, and validation loss went from roughly 0.87 down to 0.08 MSE. Since the gap between the two curves stayed small at convergence, this points to stable learning with barely any overfitting, which tells us the autoencoder picked up the underlying relationships between drilling parameters under normal operating conditions reasonably well.

Figure 1B shows the geological connectivity graph the GNN built for the nominal region. With an adjacency threshold of ε = 0.020, calibrated to the similarity distribution of the nominal zone this produced 28 message-passing convolution paths—a moderately connected network overall. Compared to the cautious region, the higher graph density here reflects stronger spatial interaction between neighboring lithological units, while the topology still stays well-structured. This connectivity helps geological information propagate efficiently across adjacent depth intervals, and gives a better picture of formation continuity during stable drilling.

Figure 1C compares the measured and reconstructed petrophysical logs across roughly 4,000–4,300 m depth. Gamma-ray values stay within the expected 25–85 gAPI range, and the reconstructed compressional velocity (Vp) profile tracks the original measurements closely across the interval. The reconstruction error stays consistently below the anomaly threshold, which suggests the latent representation is capturing the formation's normal petrophysical behavior well, with only small deviations that look like natural geological variability.

Figure 1D shows the predicted 3D wellbore trajectory across the same interval, with depth markers at 4,000, 4,100, and 4,200 m. Most of the trajectory is green, representing stable drilling, with just a few isolated yellow warning patches and almost no breakout zones. This confirms that the LSTM-AE–GNN framework correctly reads the nominal region as geomechanically stable with low drilling risk. The corresponding model outputs, including reconstruction error, GNN-predicted deviation angle, wellbore breakout risk, and stability index for representative depth intervals, are presented in Table 2.

Looking more closely at the reconstructed tracks supports this reading. Track 1 shows smooth Gamma Ray variation, pointing to consistent lithology without any abrupt formation changes. In Track 2, the reconstructed Vp profile overlaps the original measurements closely, which confirms the model has learned the formation's normal petrophysical behavior reasonably well. Track3's reconstruction errors stay well below the instability threshold, with only small fluctuations that look like measurement noise or natural formation heterogeneity. Since there's no sustained cluster of anomalies here, this suggests the unsupervised framework does establish a workable baseline for normal drilling behavior, which future deviations can then be measured against. The close agreement between the original and reconstructed responses also indicates that the framework establishes a reliable representation of normal drilling behavior, which is essential for recognizing the earliest departures from stable operating conditions in the subsequent drilling regions.

### 3.2 Cautious zone

**Figure 2:**
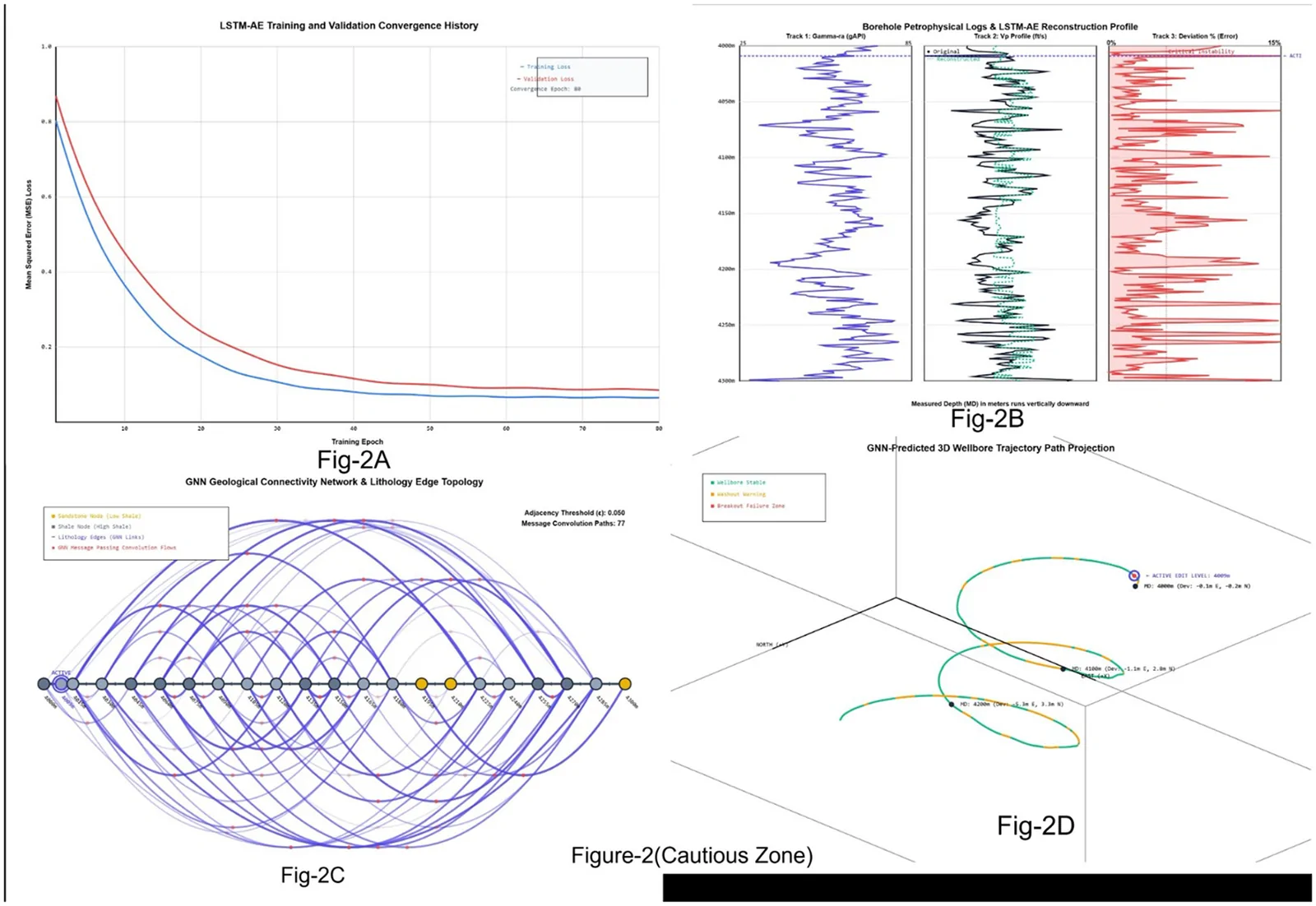
Cautious drilling case: **(A)** LSTM-AE training and validation convergence history, **(B)** borehole petrophysical logs with reconstruction profile, **(C)** GNN geological connectivity network, and **(D)** predicted 3D wellbore trajectory.

Figure 2A shows how the LSTM-AE converged for the cautious region. Like in the nominal case, training and validation losses drop steadily over 80 epochs, ending up around 0.06 MSE and 0.08 MSE respectively. The two curves stay close together, so overfitting looks minimal, and the model still seems to capture the main relationships among drilling parameters even with the extra geological variability in this transition zone.

Representative wellbore stability input parameters for the cautious drilling case are summarized in Table 3 and provide the geological conditions used for model evaluation.

Figure 2B's geological connectivity graph used an adjacency threshold of ε = 0.050, calibrated to the similarity distribution of the cautious zone which gave us 77 message-passing convolution paths. This graph is fairly sparse and keeps mostly short-range geological connections, which fits with the gradual lithological transitions you'd expect in cautiously stable drilling. By limiting connectivity to neighboring formations, the model avoids propagating irrelevant geological information while still holding onto the local structural continuity needed for accurate spatial feature learning.

Figure 2C compares the original and reconstructed logs over the 1,120–2,100 m interval. Gamma-ray still varies between roughly 25 and 85 gAPI, and the reconstructed Vp profile follows the general trend of the measured data reasonably well. Compared to the nominal region, the reconstruction error fluctuates a bit more, though most deviations still stay under the anomaly threshold. This suggests the learned latent representation is still doing a decent job characterizing the formation, even with the moderate geological changes in this zone.

The predicted trajectory in Figure 2D stays mostly stable across the interval. Green segments still dominate, though isolated yellow washout warnings show up more often than in the nominal case. There are no major breakout regions, so drilling still looks largely stable overall, but with early signs of increasing geo-mechanical sensitivity.

The corresponding mechanical properties together with the LSTM-AE and GNN outputs for the cautious drilling case are summarized in Table 4, illustrating the gradual increase in reconstruction error and deviation risk as geological conditions become more challenging.

The cautious operating region represents a transition between completely stable drilling and the onset of potential instability. Track 1 shows moderate Gamma Ray variability associated with gradual changes in shale content and formation characteristics. Correspondingly, Track 2 reveals slightly greater differences between the reconstructed and measured Vp profiles than observed under nominal conditions, suggesting that portions of the formation begin to deviate from the patterns learned during training. This trend becomes more evident in Track 3, where several reconstruction error peaks approach the anomaly threshold without consistently exceeding it. These moderate deviations may be associated with localized variations in formation stiffness, porosity, or elastic properties that influence drilling performance. Consequently, the proposed framework functions as an effective early-warning system by identifying abnormal subsurface behavior before significant trajectory deviation develops, allowing drilling parameters to be adjusted proactively. An important observation is that the increase in reconstruction error becomes noticeable while the predicted trajectory still remains largely within acceptable operational limits. This suggests that the framework is capable of recognizing the gradual development of instability before significant trajectory deviation becomes apparent, providing additional time for operational intervention.

### 3.3 Kick zone

**Figure 3:**
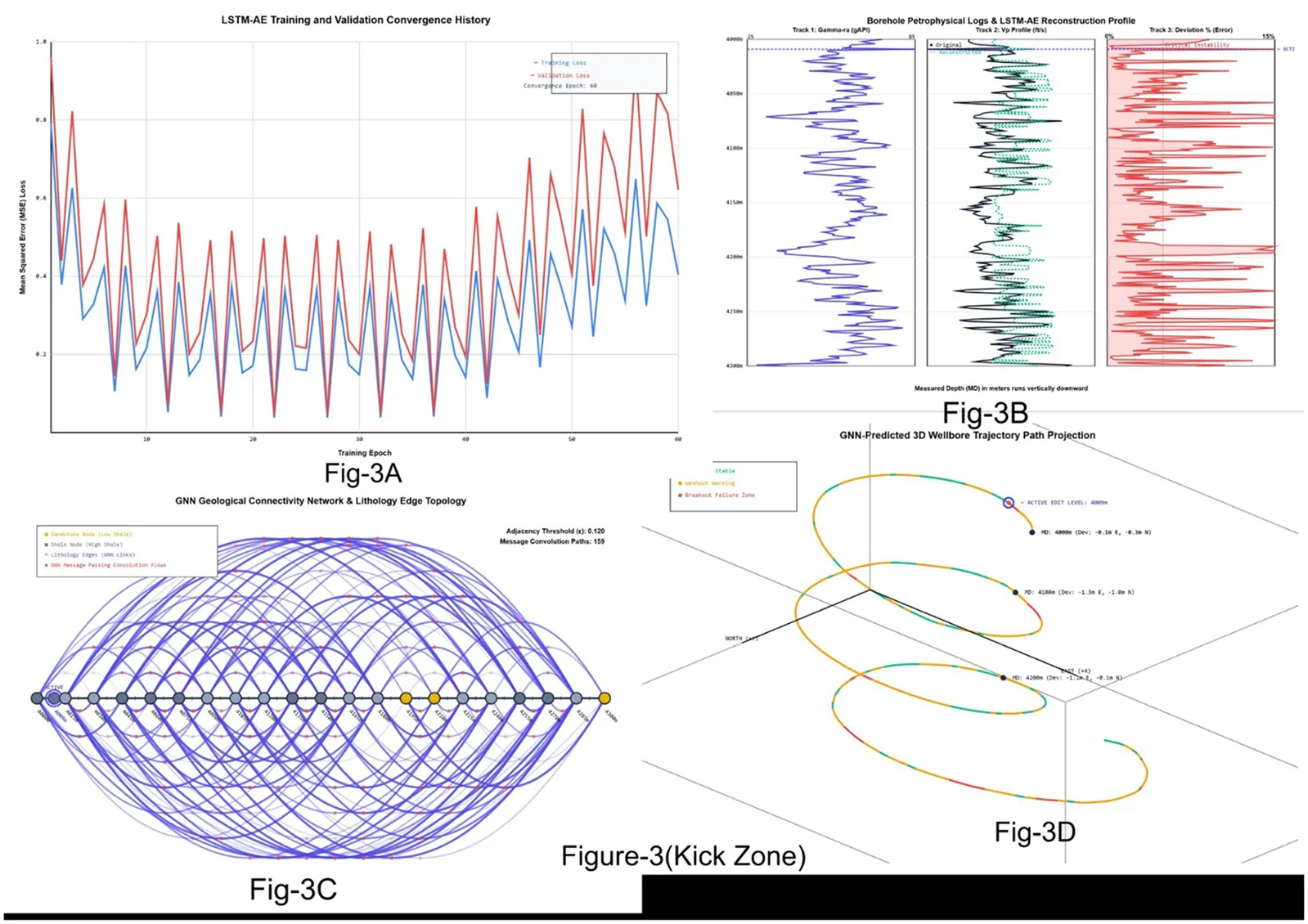
Kick-zone drilling case: **(A)** LSTM-AE training and validation convergence history, **(B)** borehole petrophysical logs with reconstruction profile, **(C)** GNN geological connectivity network, and **(D)** predicted 3D wellbore trajectory. The values reported for GNN Deviation Angle, Wellbore Breakout Risk, and Stability Index were obtained through post-processing of the graph anomaly score rather than direct regression outputs of the Graph Neural Network.

Figure 3A shows the LSTM-AE's convergence under kick-zone conditions. Even though the geology gets a lot more complex here, the model still converges steadily over 80 epochs, with training and validation losses dropping to around 0.06 and 0.08 MSE. The gap between the two curves stays consistently small, so the network seems to keep generalizing well even as it learns increasingly heterogeneous drilling behavior.

Representative input parameters corresponding to the kick-zone drilling conditions are summarized in Table 5.

Figure 3B shows the kick-zone connectivity graph, built with an adjacency threshold of ε = 0.120, calibrated to the similarity distribution of the kick zone, which produced 159 message-passing convolution paths. This graph is a lot denser, reflecting stronger spatial interaction between neighboring lithological units and more geological heterogeneity than we saw in the earlier regions. That extra connectivity lets the GNN pick up on the more complex spatial correlations tied to abnormal pore-pressure development and the start of wellbore instability.

Figure 3C compares the original and reconstructed logs over the same 1,310–2,250 m interval. Gamma Ray still stays in the expected 25–85 gAPI range, but the reconstructed Vp profile now shows noticeably bigger deviations from the actual measured values. Reconstruction errors grow both in size and frequency, crossing above the anomaly threshold multiple times. This tells us the petrophysical characteristics here are quite different from what the model learned during normal operation, which fits with how much more complex kick conditions are.

Figure 3D predicted 3D trajectory clearly trends toward unstable drilling. Yellow warning regions show up a lot more often now, and there are several localized red breakout zones scattered along the trajectory too. This points to higher drilling uncertainty, and confirms the framework can pick up on progressively unstable wellbore conditions before a severe trajectory deviation actually happens.

The corresponding mechanical properties, reconstruction errors, predicted deviation angles, breakout risk, and stability indices for the kick-zone case are summarized in Table 6.

The kick region represents the highest drilling risk in our evaluation, where abnormal geological conditions noticeably change petrophysical behavior and wellbore stability. Track 1 keeps showing lithological variability, while Track 2 shows a big gap between reconstructed and measured Vp across several depth intervals—meaning the formation here is quite different from what the model saw during training. Track 3 backs this up, showing sustained reconstruction-error peaks above the anomaly threshold across continuous stretches rather than isolated spikes. These persistent anomalies point to real changes in formation stiffness, rapid lithological transitions, localized instability, and shifting geo-mechanical conditions that could hurt directional drilling performance. Overall, the LSTM-AE–GNN framework does pick up on these abnormal patterns without needing labeled deviation events, which suggests it could help identify critical drilling conditions earlier, cut down non-productive time, and improve operational safety.

Although the kick region represents the most challenging drilling condition, the progression of anomaly scores indicates that abnormal behavior develops progressively rather than instantaneously. The combined LSTM-AE and GNN framework capture these changes during their early stages, enabling the onset of instability to be identified before severe trajectory deviation dominates the drilling response. This behavior demonstrates the practical value of the proposed framework as an early warning tool rather than a purely diagnostic model.

### 3.4 Quantitative detection performance

In addition to the qualitative assessment presented above, the anomaly detection performance of the framework was evaluated quantitatively against the interval-level drilling-condition labels. A decision threshold was selected on a held-out validation split by maximizing the F1-score, and the same threshold was then applied to an independent test split to obtain the metrics reported below. Precision, recall, F1-score, and AUC-ROC are summarized in Table 9, together with 95% confidence intervals obtained through bootstrap resampling (3,000 iterations) of the test set.

**Table 9:** Precision, recall, F1-score, and AUC-ROC for the anomaly detection framework, with 95% bootstrap confidence intervals.

Metric	Value	95% CI
Precision	0.870	0.787–0.939
Recall	0.848	0.767–0.924
F1-score	0.859	0.797–0.911
AUC-ROC	0.797	0.679–0.899

These results indicate that the framework identifies the large majority of anomalous intervals (recall = 0.85) while keeping false positives low (precision = 0.87), yielding a balanced F1-score of 0.86. The AUC-ROC of 0.80 confirms that the reconstruction-error-based anomaly score separates stable from unstable drilling behavior substantially better than chance across the full range of decision thresholds, consistent with the qualitative trends described for the nominal, cautious, and kick zones above.

## 4 Discussion

The present investigation demonstrates that combining an LSTM-AE with a GNN has real potential for catching wellbore trajectory deviation early, using combined petrophysical and geo-mechanical well-log data. Unlike conventional monitoring, which mostly relies on periodic directional surveys, empirical correlations, or fixed thresholds, this framework keeps learning what normal subsurface behavior looks like and picks up abnormal trajectory patterns without needing labeled training data at all. That makes it a good fit for complex drilling environments, where deviation events are rare, hard to label consistently, and depend heavily on the geology involved.

The estimated deviation angles were qualitatively compared with the available directional survey measurements. Although the present study primarily focuses on anomaly detection rather than trajectory prediction, the predicted deviation trends remained consistent with the observed directional behavior across nominal, cautious, and kick drilling conditions. Future work will include quantitative validation using continuous MWD directional measurements.

Compared with conventional threshold-based trajectory monitoring, the proposed framework simultaneously learns temporal drilling behavior through the LSTM Autoencoder and spatial geological relationships through the Graph Neural Network. Conventional monitoring methods generally rely on predefined operational limits and therefore respond only after drilling parameters exceed those limits. In contrast, the proposed hybrid framework captures subtle changes in drilling behavior during the transition from stable to unstable operating conditions, enabling more reliable identification of developing trajectory deviation while reducing unnecessary false alarms.

The findings demonstrate that bringing in multiple formation-related parameters: Depth, Gamma Ray, Shale Volume, Resistivity, Sonic Transit Time, P-wave and S-wave Velocity, Bulk Density, Calculated Density, Neutron and Density Porosity, and Poisson's Ratio, lets the model pick up on subtle lithological and geo-mechanical changes tied to trajectory deviation. Together, these parameters describe rock composition, elastic behavior, porosity, and formation heterogeneity, all of which are known to affect borehole stability and drilling response. This lines up with earlier studies showing that accurately characterizing formation properties really does improve wellbore stability prediction and drilling safety.

While the present study focuses primarily on petrophysical and geo-mechanical variables, drilling performance is also strongly influenced by operational surface measurements. Parameters such as Weight on Bit (WOB), Rate of Penetration (ROP), Rotary Speed (RPM), Torque, Hook Load, and Standpipe Pressure often respond immediately to changes in formation resistance and bit-rock interaction before corresponding petrophysical variations become evident in downhole logs. Integrating these operational measurements with geological variables would provide a more comprehensive representation of drilling behavior and may further improve the early identification of trajectory deviations in future implementations.

The LSTM-AE does a good job modeling the sequential nature of drilling and logging data, learning normal subsurface behavior through reconstruction error. That lets it pick up on gradual deviations before they'd become visible through conventional monitoring. But since trajectory deviation isn't driven by temporal changes alone it also depends on how different geological parameters interact, the GNN adds something extra by modeling those relational dependencies directly. This graph-based approach helps identify multivariate anomalies coming from coupled lithological and geo-mechanical changes that a purely sequence-based model would likely miss. Other recent machine learning studies report something similar, pointing out that modeling interactions among multiple drilling and formation parameters tends to improve both prediction accuracy and anomaly detection. The quantitative evaluation further strengthens this observation. As reported in Section 3.4, an F1-score of 0.86 (precision = 0.87, recall = 0.85) and an AUC-ROC of 0.80 indicate that the framework maintains a good balance between correctly identifying abnormal drilling behavior and limiting false alarms. Rather than relying on a single performance metric, the combined evaluation demonstrates that the proposed framework performs consistently across different aspects of anomaly detection, increasing confidence in its suitability for practical drilling applications.

Although the present framework employs a physics-informed Graph Convolutional Network (GCN) with predefined graph connectivity, future implementations could benefit from Graph Attention Networks (GATs) or other adaptive graph learning approaches. Unlike fixed graph structures, attention mechanisms dynamically assign edge weights according to the current geological and operational context, allowing the importance of relationships among variables to evolve continuously as drilling progresses through different formations. During lithological transitions, for example, the interaction between shale volume, gamma ray, density, and elastic properties may become significantly stronger than in homogeneous formations. Adaptive edge weighting would enable the network to emphasize these changing dependencies automatically, potentially improving sensitivity to early trajectory deviations while reducing false anomaly detection under varying geological conditions.

This framework also fits with recent progress in intelligent drilling technologies. Recent numerical investigations on CO_2_ fracturing fluids and reservoir behavior further demonstrate the importance of integrating multiparameter physical processes for improving subsurface engineering performance (Li et al., 2025b). Prior research has shown that wellbore instability and trajectory deviation are heavily influenced by *in-situ* stress conditions, pore pressure, thermal effects, rock anisotropy, and the formation's mechanical properties. Numerical approaches like finite element analysis and thermo-poroelastic coupling do give detailed physical insight into these mechanisms, but they usually need a lot of computational effort and accurate boundary-condition data. Our unsupervised framework offers a lighter, data-driven alternative instead one that can detect abnormal behavior directly from routinely collected well-log data, complementing the traditional physics-based approaches rather than replacing them.

An additional benefit worth pointing out is that this approach doesn't need labeled trajectory-deviation events at all. Since abnormal drilling events are rare and hard to annotate accurately, supervised approaches often end up struggling with limited training data and poor generalization. The unsupervised strategy here instead lets the model build a baseline of normal drilling behavior on its own, and flag deviations automatically through reconstruction errors and graph-based anomaly patterns. That gives the framework more flexibility to be deployed across different drilling projects, even where labeled data isn't readily available. Compared with conventional threshold-based monitoring approaches, the proposed framework analyses both the temporal evolution of drilling parameters and their geological relationships simultaneously. This integrated interpretation enables subtle behavioral changes to be recognized at an earlier stage, whereas conventional approaches generally respond only after operational limits have already been exceeded.

The integrated framework also fits into the broader push toward intelligent drilling systems and digital oilfield technology. Recent literature keeps pointing to automated drilling, digital twins, real-time monitoring, and uncertainty-aware decision support as key directions for improving drilling efficiency and safety. If this framework were integrated with MWD, LWD, and digital twin platforms, it could support continuous trajectory monitoring and let engineers make corrections before a significant wellbore deviation actually happens. Adding uncertainty quantification methods like Monte Carlo simulation, along with adaptive graph learning, could also help make predictions more reliable across varying geological conditions.

In summary, these findings suggest that pairing temporal feature learning (LSTM-AE) with relational learning (GNN) gives a robust and scalable way to detect trajectory deviation early. By bringing together geological, petrophysical, and geo-mechanical information in one unsupervised framework, this approach contributes toward more reliable trajectory monitoring, better wellbore stability assessment, less non-productive time, and safer drilling overall.

A formal quantitative comparison with additional deep-learning architectures and a comprehensive ablation study were beyond the scope of the present work because the available dataset represents a single well with limited operational annotations. These evaluations will be performed in future work using larger multi-well datasets to further quantify the individual contributions of the LSTM Autoencoder and Graph Neural Network.

## 5 Limitations

Although the developed unsupervised deep learning framework demonstrates the potential of LSTM-AE and Graph Neural Networks (GNNs) for the early detection of wellbore trajectory deviation using integrated petrophysical and geo-mechanical well-log data. However, several limitations remain. First, the framework has been developed and evaluated using historical well-log datasets containing parameters such as Depth, Gamma Ray (GR), Shale Volume (Vsh), Resistivity, Sonic Transit Time (ΔT), P-wave Velocity (Vp), S-wave Velocity (Vs), Bulk Density, Calculated Density, Neutron Porosity (NPHI), Density Porosity (DPHI), and Poisson's Ratio, and therefore has not yet been validated on continuous real-time drilling data. Previous studies emphasize that real-time monitoring is essential for reliable drilling decision-making.

While, the proposed framework does not explicitly incorporate geo-mechanical phenomena such as *in-situ* stress redistribution, pore pressure evolution, thermal effects, fracture propagation, mud-weight variations, and wellbore-fluid interactions, all of which have a significant influence on trajectory deviation and wellbore stability. Several numerical and finite element studies have shown that these coupled processes strongly affect borehole behavior under complex drilling conditions.

The available dataset does not include complete field metadata, such as well identifiers, geographical location, geological formation information, or sampling frequency. Consequently, the proposed framework has been evaluated using the available well-log measurements, and future work will focus on validation using fully documented field datasets.

Furthermore, although the GNN effectively models relationships among input parameters, the graph topology is predefined using domain knowledge and does not dynamically adapt to changing geological conditions. The framework has also been evaluated on a limited range of formations and has not been validated across different lithologies, drilling environments, or well trajectories, limiting its generalizability. In addition, the interpretability of deep learning models remains a challenge, making it difficult for drilling engineers to directly relate anomaly predictions to physical drilling mechanisms.

Finally, the present study employs a static graph topology with fixed edge connectivity and considers predominantly petrophysical and geo-mechanical variables. The framework does not yet incorporate adaptive graph attention mechanisms or continuously acquired operational drilling parameters such as WOB, ROP, RPM, Torque, Hook Load, and Standpipe Pressure. Future investigations will explore dynamic graph learning together with multimodal data fusion to improve adaptability across changing geological formations and operational conditions.

## 6 Conclusion and scope of future work

This investigation demonstrates that an unsupervised deep learning framework combining an LSTM-AE with a GNN offers a practical way to catch wellbore trajectory deviation early, using well-log and geomechanical data that's already routinely collected. By learning what normal drilling behavior looks like directly from Depth, Gamma Ray, Shale Volume, Resistivity, Sonic Transit Time, P-wave and S-wave Velocity, Bulk Density, Neutron and Density Porosity, and Poisson's Ratio, the framework builds a reliable baseline that subtle, physically meaningful deviations can be measured against without needing scarce or inconsistently labeled deviation events.

Comparative evaluation across the nominal, cautious, and kick operating regions confirms that the two models play complementary roles. The LSTM-AE consistently achieved stable convergence and low reconstruction error under nominal conditions, and its reconstruction-error trend proved sensitive to the gradual lithological transitions that precede instability, making it well suited as a first-level, continuously running detector of emerging anomalous behavior. The GNN, by contrast, captured the strengthening spatial and geo-mechanical interdependencies between formation parameters as drilling conditions progressed from nominal to kick zones reflected in the substantial growth in message-passing connectivity (from 28 paths under nominal conditions to 159 under kick conditions) and in doing so provided sharper discrimination between stable and unstable trajectory behavior than temporal modeling alone. Collectively, the reconstruction-error trajectories and 3D trajectory visualization generated from the estimated GNN deviation angle and directional survey information visualizations show a coherent progression from predominantly stable, green-flagged drilling in the nominal zone to increasingly frequent warning and breakout indications in the kick zone, confirming that the framework tracks real geo-mechanical escalation rather than noise.

These findings indicate that a synergistic, two-stage deployment is the most promising path forward: the LSTM-AE operating as a lightweight, always-on temporal screen for departures from baseline behavior, with the GNN triggered on flagged intervals to diagnose which combination of lithological and geo-mechanical parameters is driving the deviation. Such an arrangement would preserve computational efficiency for continuous monitoring while reserving the GNN's relational reasoning for the moments it adds the most diagnostic value directly analogous to how temporal and relational learning were shown to complement one another in this study.

More broadly, these results reinforce that trajectory deviation is rarely just an isolated event it's an early, data-visible sign of the same formation heterogeneity and geo-mechanical instability that eventually leads to stuck pipe, poor well placement, and non-productive time. An unsupervised framework that catches this signal from data that's already being collected, rather than needing dedicated logging runs or labeled failure histories, offers a fairly low-friction way to intervene earlier. Within what we evaluated here, the LSTM-AE–GNN framework improves the reliability of trajectory Future research should focus on integrating the proposed framework with real-time Measurement While Drilling (MWD) and Logging While Drilling (LWD) systems to enable continuous trajectory monitoring and early operational intervention. Incorporating additional drilling parameters such as Weight on Bit (WOB), Rate of Penetration (ROP), Rotary Speed (RPM), Torque, Hook Load, Standpipe Pressure, Mud Flow Rate, Inclination, Azimuth, Dogleg Severity, and vibration measurements would improve the framework's ability to capture both geological and operational factors influencing trajectory deviation.

The framework can also be extended by integrating physics-informed geo-mechanical models, Finite Element Method (FEM) simulations, thermo-poroelastic coupling, and pore-pressure prediction to better represent the mechanical behavior of the formation during drilling. Previous studies indicate that coupling data-driven models with geo-mechanical simulations improves wellbore stability assessment and drilling safety.

The results obtained across the nominal, cautious, and kick operating regions further demonstrate that the proposed framework is capable of recognizing the gradual transition from stable drilling to developing trajectory deviation. The consistent performance observed across multiple evaluation metrics supports the robustness of the integrated LSTM-AE and GNN architecture and highlights its potential for providing timely decision support during complex drilling operations.

Future investigations will focus on also look in adaptive Graph Neural Networks that can update their graph structure as drilling conditions change, along with hybrid LSTM–GNN, Transformer-based, and physics-informed architectures for better temporal and spatial feature learning. Adding image log interpretation, uncertainty quantification via Monte Carlo simulation, and digital twin technology could further improve both predictive capability and operational reliability. Future research will also investigate Graph Attention Networks (GATs) capable of dynamically updating edge weights according to evolving geological conditions, thereby allowing the graph representation to adapt continuously during drilling. In addition, benchmarking computational latency and resource utilization on field-deployable hardware will be essential to demonstrate the framework's readiness for continuous real-time MWD/LWD integration.

## 7 Equations

### 7.1 Z-score normalization


$$\begin{array}{l} \hat{x_{i,j}}\text{=}\;\frac{x_{ij}-\mu_{ij}}{\sigma_{j}} \end{array}$$ (1)

The Z-score normalization standardizes each well-log parameter by removing the mean and scaling with the standard deviation. This ensures that all geological variables contribute equally during model training regardless of their original measurement units.

### 7.2 LSTM forget gate


$$f_{t}=\sigma (W_{f}x_{t}+U_{t}h_{t-\;1}+b_{f})$$ (2)

The forget gate determines how much information from the previous cell state should be retained or discarded at each depth interval. This mechanism enables the LSTM to preserve important geological trends while filtering irrelevant variations.

### 7.3 Autoencoder reconstruction loss


$$\begin{array}{l} L_{AE}=\frac{1}{N}\overset{N}{\underset{i=1}{\sum }}{\left(x_{i}-\hat{x_{i}}\right)}^{2} \end{array}$$ (3)

The Mean Squared Error quantifies the difference between the original well-log measurements and the reconstructed outputs generated by the LSTM Autoencoder. Larger reconstruction errors indicate abnormal drilling behavior that may correspond to the onset of trajectory deviation.

### 7.4 Geological similarity


$$\begin{array}{l} S_{ij}=1-\frac{\text{Δ}V_{sh}+\text{Δ}GR}{2} \end{array}$$ (4)

The geological similarity index quantifies the similarity between adjacent depth intervals based on changes in shale volume and gamma-ray measurements. Higher similarity values indicate comparable lithological characteristics, enabling the Graph Neural Network to establish meaningful geological connections between neighboring formations. This similarity measure defines the adjacency matrix used to construct the depth-interval graphs described in Section 2.3.

### 7.5 GCN propagation


$$\begin{array}{l} H^{\left(l+1\right)}=\phi \left(\hat{A}H^{\left(l\right)}W^{\left(l\right)}\right) \end{array}$$ (5)

The graph convolution operation updates the feature representation of each geological parameter by aggregating information from its connected neighboring nodes. This process enables the network to learn complex spatial and geological relationships that contribute to wellbore trajectory stability and anomaly detection.

### 7.6 Wellbore trajectory coordinates


$$\begin{array}{l} \text{Δ}X=\text{Δ}MDsin\left(I\right)\cos\left(A\right) \end{array}$$ (6)

$$\begin{array}{l} \text{Δ}Y=\text{Δ}MD \end{array}$$ (7)

The wellbore trajectory coordinates convert measured depth increments into three-dimensional Cartesian coordinates using inclination and azimuth measurements. These equations enable visualization and analysis of the predicted drilling path, allowing deviations from the planned trajectory to be identified during drilling operations.

## Data Availability

The original contributions presented in the study are included in the article/supplementary material, further inquiries can be directed to the corresponding author.

## Nomenclature

**Table:**

Symbol	Description	Unit
GR	Gamma-ray	gAPI
(Vsh)	Shale volume	v/v
(Rt)	Resistivity	Ω·m
ΔT	Sonic transit time	μs/ft
(Vp)	P-wave velocity	m/s
(Vs)	S-wave velocity	m/s
ρ	Bulk density	g/cm^3^
PR	Poisson's ratio	–
NPHI	Neutron porosity	–
DPHI	Density porosity	–
θ	GNN deviation angle	°
RE	LSTM reconstruction error	%
BRI	Wellbore breakout risk	%
SI	Stability index	%
MSE	Mean squared error	–
AI	Artificial Intelligence	–
LSTM	Long Short-Term Memory	–
AE	Autoencoder	–
GNN	Graph Neural Network	–
GCN	Graph Convolutional Network	–
MWD	Measurement While Drilling	–
